# HDAC9 is an epigenetic repressor of kidney angiotensinogen establishing a sex difference

**DOI:** 10.1186/s13293-017-0140-z

**Published:** 2017-05-30

**Authors:** Camille T. Bourgeois, Ryousuke Satou, Minolfa C. Prieto

**Affiliations:** Department of Physiology, Tulane Hypertension and Renal Center of Excellence, Tulane University School of Medicine, 1430 Tulane Avenue, SL39, New Orleans, LA 70112-2699 USA

**Keywords:** Histone deacetylase 9, Angiotensinogen, Kidney, Sex differences, Renin-angiotensin system, Epigenetics

## Abstract

**Background:**

Sexual difference has been shown in the pathogenesis of chronic kidney disease induced by hypertension. Females are protected from hypertension and related end-organ damage. Augmentation of renal proximal tubular angiotensinogen (AGT) expression can promote intrarenal angiotensin formation and the development of associated hypertension and kidney injury. Female rodents exhibit lower intrarenal AGT levels than males under normal conditions, suggesting that the suppressed intrarenal AGT production by programmed mechanisms in females may provide protection from these diseases. This study was performed to examine whether epigenetic mechanisms serve as repressors of AGT.

**Methods:**

Male and female Sprague Dawley rats were used to investigate sex differences of systemic, hepatic, and intrarenal AGT levels. All histone deacetylase (HDAC) mRNA levels in the kidneys were determined using a PCR array. HDAC9 protein expression in the kidneys and cultured renal proximal tubular cells (PTC) was analyzed by Western blot analysis and immunohistochemistry. The effects of HDAC9 on AGT expression were evaluated by using an inhibitor and siRNA. ChIP assay was performed to investigate the interaction between the AGT promoter and HDAC9.

**Results:**

Plasma and liver AGT levels did not show differences between male and female Sprague-Dawley rats. In contrast, females exhibited lower AGT levels than males in the renal cortex and urine. In the absence of supplemented sex hormones, primary cultured renal cortical cells isolated from female rats sustained lower AGT levels than those from males, suggesting that the kidneys have a unique mechanism of AGT regulation controlled by epigenetic factors rather than sex hormones. HDAC9 mRNA and protein levels were higher in the renal cortex of female rats versus male rats (7.09 ± 0.88, ratio to male) while other HDACs did not exhibit a sex difference. HDAC9 expression was localized in PTC which are the primary source of intrarenal AGT. Importantly, HDAC9 knockdown augmented AGT mRNA (1.92 ± 0.35-fold) and protein (2.25 ± 0.50-fold) levels, similar to an HDAC9 inhibitor. Furthermore, an interaction between HDAC9 and a distal 5’ flanking region of AGT via a histone complex containing H3 and H4 was demonstrated.

**Conclusions:**

These results indicate that HDAC9 is a novel suppressing factor involved in AGT regulation in PTC, leading to low levels of intrarenal AGT in females. These findings will help to delineate mechanisms underlying sex differences in the development of hypertension and renin-angiotensin system (RAS) associated kidney injury.

## Background

Women and female rodents have significantly lower systolic and diastolic blood pressures (BP) than male counterparts [1] and are protected from the development of hypertension and related end-organ damage compared to males [2–4]. The renin-angiotensin system (RAS) is critical in the control of BP and the regulation of electrolyte and body fluid homeostasis [5]. In the systemic RAS, angiotensinogen (AGT), the precursor of angiotensin II (Ang II), is mainly produced in the liver and determines levels of angiotensin formation in plasma and BP [6, 7]. Sex hormones influence both plasma and hepatic AGT levels [8], and systemic AGT levels in females are thought to be greater than males because of estrogen stimulation. Estrogen-responsive elements are located on the AGT promoter [9], and estrogen administration stimulates AGT production in the liver of female rats [8]. Thus, higher expression levels of liver AGT and concentration of plasma AGT in females than males are expected. However, there are no differences in either plasma or hepatic AGT levels between adult males and females under normal conditions [10, 11]. Furthermore, stimulation of AGT expression by estrogen may not explain previous findings that females exhibit resistance against hypertension and RAS-induced tissue injuries.

The presence of a tissue RAS has been established in individual organs and functions in a tissue-specific manner [7, 12]. Since intrarenal Ang II levels are elevated in many forms of hypertension, the intrarenal RAS is a key target for studies associated with hypertension and kidney injury [13]. Indeed, the elevation of intrarenal Ang II is associated with the augmentation of intrarenal AGT, which is primarily produced in renal proximal tubular cells (PTC) [14, 15]. Renal proximal tubule-specific overexpression of AGT amplifies intrarenal Ang II levels and promotes the development of hypertension and kidney injury in male mice [16, 17]. Intrarenal AGT expression levels in female rodents are lower than in males [11, 18]. Therefore, a sex difference in intrarenal AGT production is a potential mechanism for delineating the pathophysiological resistance of females against the development of RAS-associated diseases. However, sex differences in the mechanisms underlying intrarenal AGT regulation remain unclear.

Histone deacetylases (HDACs) are enzymes that repress gene expression through the removal of acetyl groups from histones [19]. HDACs play a role in the regulation of BP and end-organ damage [20] and have been linked to sex differences in both liver and kidney injury [21, 22]. Inhibition of class I HDACs (HDAC1, 2, and 3) reduces pulmonary arterial pressure, indicating that class I HDACs are risk factors for the development of hypertension in male rats [23]. In contrast, class IIa HDACs (HDAC4, 5, 7, and 9) attenuate the development of Ang II-induced cardiac hypertrophy [24]. Because both ovariectomy in female rats and castration in male rats do not alter basal AGT expression levels in the kidneys of non-salt-loaded Dahl rats [18], we hypothesized that epigenetic repressors such as HDACs limit intrarenal AGT expression in females and could at least in part explain the sex disparities in hypertension and renoprotection. Although physiological and pathological roles for the RAS and its regulation have been studied extensively for many decades, the interplay between epigenetic factors and the RAS is still in the early stages of discovery. In particular, epigenetic regulation of intrarenal AGT has not been established. In the present study, we used in vivo and in vitro approaches to elucidate the epigenetic mechanisms regulating intrarenal AGT expression, leading to sex differences in AGT originating from the proximal tubule.

## Methods

### Animal and tissue samples

All protocols were evaluated and approved by the Tulane Institutional Animal Care and Use Committee and conformed to the guidelines of the National Institutes of Health on the care and use of laboratory animals. Male and female Sprague-Dawley rats, 7 weeks of age (Charles River Laboratories), were cage-housed and maintained in a temperature-controlled room on a 12-h light to dark cycle, with free access to tap water and rat chow during acclimation. Twenty-four hour urine samples were collected in metabolic cages. Rats were euthanized by conscious decapitation, and trunk blood, liver, and kidney tissue were collected.

### Antibodies

A rabbit anti-histone deacetylase 9 (HDAC9) antibody from Abcam (ab109446), rabbit anti-AGT antibody from IBL (JP28101), rabbit anti acetyl-Histone H3 (Lys5, #9675), and rabbit anti-acetyl-Histone 4 (Lys18, #8647) from Cell Signaling Technology were used. A mouse anti-β-actin antibody from Abcam (ab6276) was used as an internal control. IRDye-labeled anti-mouse IgG and anti-rabbit IgG antibodies were obtained from Li-Cor (P/N925-68070 and P/N925-32211, respectively) as secondary antibodies in Western blot analyses. Alexa Fluor 488 goat anti-rabbit IgG (H + L) antibody from Life Technologies (A-11008) was used as a secondary antibody in immunostaining.

### Cell culture

Immortalized rat PTC were kindly provided by Dr. Ingelfinger (Harvard Medical School) and used in this study [25]. The cells were cultured in DMEM medium (Invitrogen) supplemented with 10% heat-inactivated fetal bovine serum (FBS) (Invitrogen) and were plated in 12-well plates. Primary cultured hepatocytes isolated from male and female Sprague-Dawley rats were purchased from Invitrogen. The hepatocytes were cultured according to the provider’s instructions. Briefly, the cells were seeded onto collagen coated flasks and cultured with Williams Medium E medium containing FBS and insulin/transferrin selenium for at least 3 days. Renal cortical cells were isolated from male and female renal cortices as previously described. In brief, the cells were separated by sieving using 212 μm metal mesh, then a single cell suspension was created using Collagenase Type I (Invitrogen). A 74-μm metal mesh was used to remove aggregating cells and tissues. Cells were cultured in DMEM medium with 10% FBS for at least 3 days. AGT expression in isolated renal cortical cells was 9% lower than renal cortical tissue samples from Sprague-Dawley rats, suggesting that the isolated cells sustained their characteristics in AGT expression during culture.

### AGT ELISA

AGT levels in urine, plasma, and cell culture medium were measured as previously described [26] using the Rat Total Angiotensinogen Assay Kit (IBL). Each urinary AGT level was normalized based on 24-h urine volume and body weight.

### Epigenetic chromatin modification enzymes PCR array

Twenty nanograms total RNA isolated from renal cortex of male and female rats was used in a PCR array. The total RNA was converted to complementary DNA (cDNA) using an RT2 First Strand Kit (SABiosciences). Rat Epigenetic Chromatin Modification Enzymes PCR array (SABiosciences), a quantitative PCR method, was employed to screen for differentially expressed HDACs and related gene transcripts between male and female renal cortex. Furthermore, messenger RNA (mRNA) expression levels of 24 methyltransferases were also analyzed in these samples. The PCR array was performed using Mx3005p (Stratagene). All values were normalized based on β-actin expression levels.

### Quantitative real-time RT-PCR

Quantitative real-time RT-PCR (qRT-PCR) was performed to evaluate rat AGT mRNA expression using the TaqMan PCR system as previously described [27]. For total RNA isolation, tissues and cells were washed with 3 ml of PBS. PBS was aspirated, and total RNA was isolated from the cells using the RNeasy Mini Kit (Qiagen). Subsequently, qRT-PCR was performed. The data were normalized based on expression levels of rat β-actin mRNA.

### Western blot analysis

AGT and HDAC9 protein levels were determined using Western blot analysis. The Western blots were performed as previously described [27, 28]. Tissues and cells were homogenized with 60 μl lysis buffer containing 1% Triton X-100, 150 mmol/l NaCl, 1 mmol/l EDTA, 1% Nonidet P-40, 1 mmol/l Na_3_VO_4_, and 0.25% Protease Inhibitor Cocktail (Sigma). The lysates were sonicated 3 times for 10 sec each. Total protein concentration of the supernatant was quantified using Micro BCA Protein Assay Kit (Pierce). Then, 20 μg of total protein was applied to a pre-cast NuPAGE 4–12% gel (Invitrogen). The separated proteins were transferred to a nitrocellulose membrane (Bio-Rad). After incubation of the membrane with primary and secondly antibodies, detection and analysis were performed using the Odyssey System (Li-Cor). Data were normalized based on rat β-actin protein expression levels. The specificity of the anti-AGT antibody and molecular size of detected bands in Western blot analysis have been shown in previous study [29, 30]. The specificity of the anti-HDAC9 antibody is demonstrated in Fig. 5.

### Immunohistochemical studies

In addition to immunoblotting, the expression of HDAC9 protein in renal cortex and PTC was confirmed by immunostaining in PTC and 3-μm paraffin-embedded rat kidney sections. PTC were cultured in 4-well chambers (Lab-Tek). The cells were rinsed with PBS and then fixed for 20 min by 4% paraformaldehyde. After 4 min incubation with 0.2% Triton X-100, the blocking agent Image-iT FX signal enhancer (Invitrogen) was added to the chambers. The cells were incubated with HDAC9 antibody overnight at 4 °C. After washing with PBS, the cells were incubated with an Alexa Fluor 594-labeled secondary antibody. Vectashield HardSet mounting medium with DAPI from Vector Laboratories was used as a nuclear stain and a mounting reagent. The HDAC9 staining was observed and photographed using a fluorescence Nikon Eclipse 50i microscope. A similar staining protocol was used in the kidney sections.

### Inhibition of HDAC9 by an inhibitor and knockdown by RNA interference technique

The role of HDAC9 in AGT expression was examined using 5 μM TMP269 (Cellagen Technology), an inhibitor of class IIa HDACs showing higher affinity to HDAC9 [31], and small interference RNA (siRNA) technology as previously described [28]. PTC were plated on 12-well plates with Lipofectamine RNAiMax (Life Technologies) containing rat negative control-siRNA (Ambion, AM4635) or HDAC9-siRNA (Ambion, sense sequence; 5’-CCC TGA CGG TAG ATG TGG ATT-3’). The negative control siRNAs have been designed to have no significant sequence similarity to mouse, rat, or human transcript sequences and tested in the industry using multiple cell lines and shown to have no significant impact on cell proliferation, apoptosis, or cell morphology. The final concentration of the siRNAs was 50 nM. OPTI-MEM I medium (Invitrogen) was used for these transfections. After 24 h transfection of siRNA, cells were harvested to determine suppression of HDAC9 protein expression using Western blot analysis. A separate group of cells was also used to evaluate the contribution of HDAC9 to the regulation of AGT expression by qRT-PCR and ELISA.

### ChIP assay

Primary cultured renal cortical cells isolated from Sprague-Dawley rats were used in the ChIP assay. The cells were cultured in DMEM medium (Invitrogen) supplemented with 10% heat-inactivated FBS. To avoid change in characteristics, the cells were used within one passage. ChIP assay was performed as previously described [32]. In the assay, an anti-HDAC9 antibody and two sets of primers designed for a distal AGT promoter region (from −1427 to −1879, forward primer: 5’-TCA GAC AGC CTT AGT AGC AA-3’, reverse primer: 5’-TGA GAA GTC TGG GAG ATG AA-3’) and a proximal AGT promoter region (from −437 to −951, forward primer: 5’- CCA GCT CAG ACA CCA TCA AA-3’, reverse primer: 5’- ACG ACC TTG AAT GGT TGT AA-3’) were used. ChIP assays were performed using anti-H3 and H4 antibodies.

### Statistical analysis

Data are expressed as means ± SE. The data were analyzed using Student *t* test. A value of *P* < 0.05 was considered statistically significant.

## Results

### Plasma and liver AGT levels in male and female rats

**Fig. 1:**
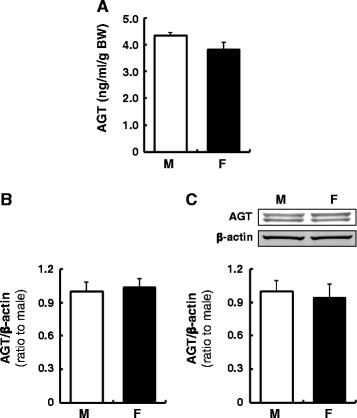
Plasma and liver AGT levels in male and female rats. Plasma AGT (**a**), liver AGT mRNA (**b**), and liver AGT protein levels (**c**) in male (*M*) and female (*F*) rats were determined by AGT ELISA, qRT-PCR and Western blot analysis, respectively. Plasma AGT concentrations were normalized based on body weights because of different body weights between age-matched male and female rats. Data are expressed as mean ± SE

There were no sex differences in plasma AGT levels (Fig. 1a, *N* = 4 in each sex). Since the primary source of plasma AGT is the liver, hepatic AGT expression levels were determined. There were no differences in liver AGT mRNA and protein expression between males and females (Fig. 1b, c, *N* = 4).

### Renal and urinary AGT levels in male and female rats

**Fig. 2:**
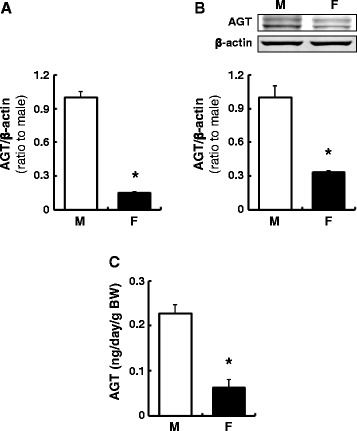
Renal and urinary AGT levels in male (*M*) and female (*F*) rats. Renal cortical AGT mRNA (**a**) and protein (**b**) levels were evaluated by qRT-PCR and Western blot analysis, respectively. AGT levels in 24-h urine samples were evaluated by AGT ELISA (**c**). Data are expressed as mean ± SE. *Asterisk* (*P* < 0.05) indicates significant difference compared with the male group

In contrast to the liver, renal cortical and urinary AGT levels showed significant sex differences. AGT mRNA and protein levels were lower in the renal cortex of female rats (Fig. 2a, b, mRNA; 0.14 ± 0.01, protein; 0.33 ± 0.01, ratio to male, *N* = 4). Furthermore, lower urinary AGT levels were observed in females compared with males (Fig. 2c, 0.23 ± 0.02 ng/day/g BW in males vs. 0.06 ± 0.02 ng/day/g BW in females, *N* = 4).

### Sex differences in AGT levels in primary cultured hepatocytes and renal cortical cells

**Fig. 3:**
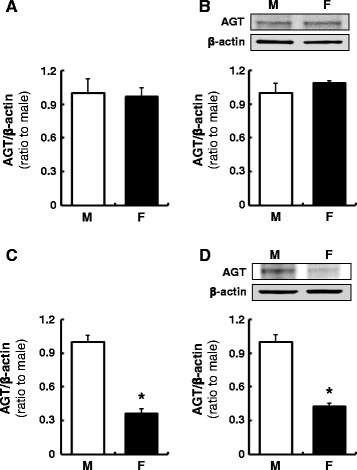
Sex differences in AGT levels in primary cultured hepatocytes and renal cortical cells. AGT mRNA and protein expression levels were evaluated in the absence of supplemented sex hormones using primary cultured hepatocytes (**a**, **b**) and renal cortical cells (**c**, **d**) isolated from male (*M*) and female (*F*) rats. Data are expressed as mean ± SE. *Asterisk* (*P* < 0.05) indicates significant difference compared with the cells isolated from male rats

AGT expression levels were evaluated using primary cultured hepatocytes and renal cortical cells isolated from male and female rats. No sex difference was observed in AGT mRNA (Fig. 3a) and protein (Fig. 3b) levels in hepatocytes. Cortical cells from females sustained lower AGT mRNA (Fig. 3c, 0.36 ± 0.04, ratio to cells isolated from males, *N* = 4) and protein (Fig. 3d, 0.43 ± 0.03, ratio to cells isolated from males, *N* = 4) levels after more than 3 days of culture.

### HDACs, their co-factor and methyltransferase levels in renal cortex of male and female rats

**Fig. 4:**
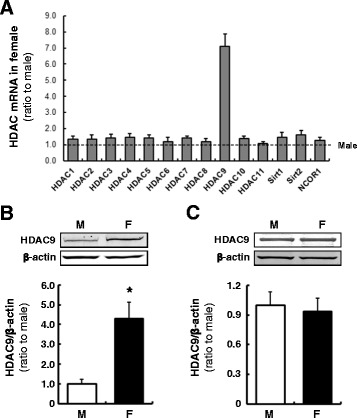
HDACs and the co-factor levels in renal cortex of male and female rats. Transcript levels of HDACs and NCOR1, a co-factor of HDACs, in the renal cortexes were determined by a real-time PCR array (**a**). Protein levels of the epigenetic factors in the kidneys (**b**) and HDAC9 protein levels in the liver (**c**) of male (*M*) and female (*F*) rats were measured by Western blot analyses. Data are expressed as mean ± SE. *Asterisk* (*P* < 0.05) indicates significant difference compared with the male group

Since renal cortices exhibited a sex difference in AGT expression, mRNA levels of HDACs including Sirt1, Sirt2, and NCOR1, a co-factor of HDACs, were determined by a real-time PCR array. HDAC9 exhibited high expression levels in the renal cortex of female rats (Fig. 4a, 7.10 ± 0.76, ratio to male, *N* = 4), while other HDACs and the co-repressor showed no changes. HDAC9 protein levels were also higher in the renal cortex of female rats (Fig. 4b, 4.31 ± 0.70, ratio to male, *N* = 4), indicating an inverse correlation between AGT and HDAC9 expression in male and female kidney cortices. This sex difference in HDAC9 expression was not observed in the liver (Fig. 4c). mRNA levels of 24 methyltransferases were also compared in renal cortex of male and female rats, and many of these enzymes (Ash2l, Cxxc1, Dot1, Edf1, Eed, Ehmt1, Ehmt2, Ezh2, Fbxo1, Men1, Mll1, Mll2, Mll5, Prdm2, Prmt1, Prmt2, Prmt5, Prmt6, Prmt7, Setdb2, Suv39h1, and Suv39h2) did not show sex differences. Renal cortical Smyd1 and Smyd3 mRNA levels in female rats were higher than male rats (1.70 ± 0.28 and 1.71 ± 0.30, respectively, ratio to male, *N* = 4).

### Localization of HDAC9 in renal cortex

**Fig. 5:**
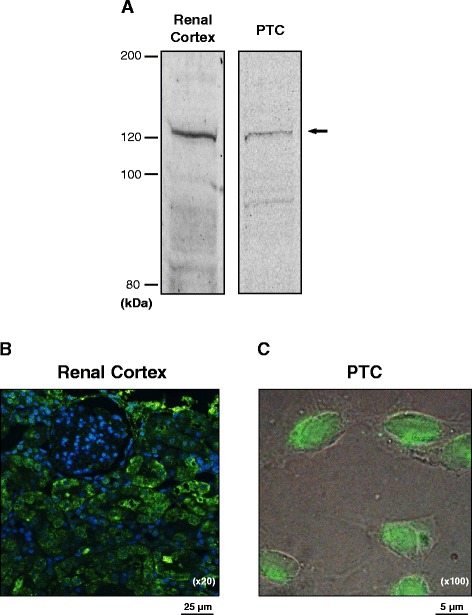
HDAC9 expression in PTC. HDAC9 expression and localization in PTC were investigated by Western blot analyses (**a**) and immunocytochemistry (**b**, **c**). In the immunocytochemistry of HDAC9 in renal cortex (**b**, ×20) and cultured PTC (**c**, ×100), immunoreactive proteins against an anti-HDAC9 antibody were visualized by a FITC-conjugated secondary antibody (*green*). Renal cortexes used in these experiments were obtained from female rats

Intrarenal AGT is mainly produced by PTC. Thus, expression of HDAC9 in PTC was tested by Western blot and immunocytochemistry. Western blot of PTC and renal cortex lysates showed immunoreactive HDAC9 bands at the expected molecular size (approximately 120 kDa), suggesting that the renal cortex and PTC express HDAC9 (Fig. 5a). Furthermore, immunostaining demonstrated that HDAC9 is localized to renal cortical tubules (Fig. 5b) and specifically the nuclear and perinuclear region of PTC (Fig. 5c), supporting previous findings of HDAC9 localization in other cells [33].

### Role of HDAC9 in AGT expression in PTC

**Fig. 6:**
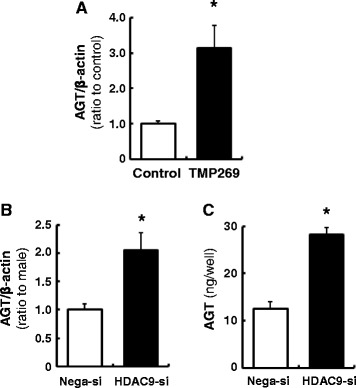
Contribution of HDAC9 to AGT expression in PTC. PTC were treated by HDAC9 inhibitor (**a**) or siRNA (**b**, **c**). Thereafter, AGT mRNA levels in the cells and AGT protein levels in the cultured medium were measured by qRT-PCR and AGT ELISA, respectively. Total AGT protein amount secreted from the cells to the culture medium were calculated based on the volume of medium. *Nega-si* negative control siRNA-transfected group, *HDAC9-si* HDAC9 siRNA-transfected group. Data are expressed as mean ± SE. *Asterisk* (*P* < 0.05) indicates significant difference compared with the negative siRNA transfected group

To investigate the function of HDAC9 in AGT regulation, PTC were treated with a class II HDAC inhibitor for 6 h. The inhibitor increased AGT expression in PTC (Fig. 6a, 3.13 ± 0.64, ratio to control, *N* = 4). Although the inhibitor exhibits a higher affinity for HDAC9, other class II HDACs are also inhibited. Thus, RNA interference using a HDAC9-specific siRNA was employed. Transfected siRNA suppressed HDAC9 levels to 31 ± 0.04% (69% knockdown efficiency, *N* = 4) in PTC. HDAC9 knockdown resulted in augmentation of AGT mRNA levels compared with control siRNA-transfected cells (Fig. 6b, 2.05 ± 0.31, ratio to control, *N* = 4). In addition, AGT protein levels in the culture medium was elevated in HDAC9-deficient cells (Fig. 6c, 12.5 ± 1.57 ng/well in the control vs. 28.2 ± 0.79 ng/well in HDAC9 siRNA-transfected cells).

### Association between HDAC9 and AGT promoter

**Fig. 7:**
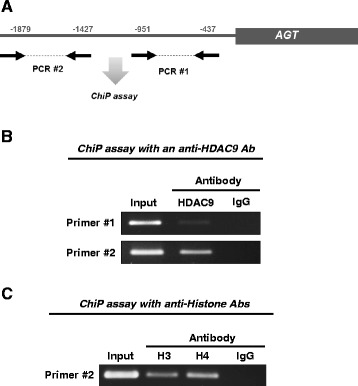
Association of HDAC9 with AGT promoter. ChiP assays were performed to test interaction between HDAC9 and AGT promoter region. Two primer sets for a proximal region (from −437 to −951 bp) and a distal region (from −1,427 to −1,879 bp) of AGT promoter were designed and used in the assay (summarized in panel **a**). An anti-HDAC9 antibody (**b**) and anti-histone H3 and H4 antibodies (**c**) were used in the assays. Rabbit IgG was used as a negative control

The interaction between HDAC9 and the AGT promoter region in rat cortical cells was investigated using ChiP assays. Two primer sets for the proximal region (−437 to −951 bp) and distal region (−1427 to −1879 bp) of the rat AGT promoter were used in the assays (Fig. 7a). After immunoprecipitation by an anti-HDAC9 antibody, the PCR product was detected using the primer for the distal region (Fig. 7b) but not the proximal region. Immunoprecipitation with IgG did not show any PCR product. The same primer set for the distal region was used to co-precipitate histones H3 and H4 and the AGT promoter. This assay indicated that histones H3 and H4 are located at the distal region of the AGT promoter (Fig. 7c).

## Discussion

Renin is the rate-limiting enzyme in the RAS; however, systemic AGT derived from the liver is also an important factor in determining levels of systemic angiotensin formation [6]. In the kidney, augmented proximal tubular AGT increases with RAS activity, leading to high BP and kidney injury as shown in renal proximal tubule-specific AGT overexpression animals [16, 17]. Although many studies have reported sex differences in RAS components due to female protection from the development of hypertension and related end-organ damage [34–38], the effects of sex hormones on liver and systemic AGT regulation remain unclear and divergent. Roles for androgen in intrarenal AGT upregulation have been tested in males [18]. Although androgen has overt stimulating effects on intrarenal AGT expression, castration did not decrease renal cortical AGT levels in male rats under normal conditions [18]. In intact and ovariectomized female rats, there were no sex differences in levels of either plasma AGT or hepatic AGT mRNA [10, 11]. However, stimulation of liver AGT production has been reported in response to estrogen administration [8], as well as in postmenopausal compared with premenopausal women [39]. A potential reason for these inconsistent results may be variations in AGT measurements which can be affected by renin activity in plasma and target tissues when an Ang I-conversion assay is used. Indeed, technical difficulties of Ang I-conversion assays to accurately determine renin activity or AGT concentration have been reported [40]. In the present study, levels of AGT in plasma, liver, and kidney were determined by ELISA which can detect both intact AGT and renin-cleaved AGT, as well as by Western blot analyses and real-time qRT-PCR using kidney cortices from age-matched male and female rats. Plasma AGT protein and liver AGT mRNA and protein levels did not show a difference between male and female under normal conditions. Similarly, no sex differences in AGT were observed in primary cultured male and female rat hepatocytes, suggesting that liver AGT is constitutively expressed and is independent of epigenetic modulation. Thus, systemic and liver AGT is unlikely to contribute to sex disparities in hypertension. Levels of AGT transcript and protein inside the kidney and the urine of female rats were lower than in males, supporting previous findings [18, 41]. The sex differences of kidney AGT mRNA and protein and urinary AGT were 3–5-fold. It has been reported that kidney AGT levels were increased 1.7-fold in Ang II-infused hypertensive rats [42]. Thus, the sex differences may have physiologically or pathologically significance. Although male and female cells were cultured in identical conditions, primary cultured renal cortical cells isolated from female rats showed lower levels of AGT mRNA and protein expression than those from males. This suggests that epigenetic regulation can contribute to establish sex differences in AGT expression in the kidney. In addition to this primary mechanism, a slight attenuation of sex differences in AGT expression was observed in primary cultured cortical cells compared with tissue samples, possibly due to the differences in physiological versus in vitro levels of sex hormones.

It has been proposed that a fraction of plasma AGT is filtered and internalized into renal proximal tubules, thus constituting the major source of intrarenal AGT, even under normal conditions [43, 44]. In the present study, we showed that intrarenal and urinary AGT protein levels were lower in females which correlated with the sex differences observed in intrarenal AGT mRNA levels. The fact that systemic AGT levels did not show these differences suggests that AGT originating from the liver is the cause of the differences observed. Moreover, there may be a kidney-specific AGT regulating mechanism that is impacted by sex.

Sex hormones and intracellular signal transducers including upstream stimulatory factors 1 and 2 have been shown to cause sex differences in local AGT production [18, 45–47]. However, epigenetic regulatory mechanisms of AGT transcription, especially in the kidney, have not been established despite the epigenome being a critical factor in BP control and the development of tissue injury [20, 48, 49]. Data in the present study indicate that HDAC9 expression in the renal cortex was higher in female rats than in male rats. Moreover, HDAC9 expression was observed in PTC. In cardiomyocytes, estrogen contributes to the retention of high levels of HDAC4 and HDAC5 which are class IIa HDACs, as well as HDAC9 [24]. The effect of estrogen on class IIa HDACs expression may explain high levels of HDAC9 in renal cortex of females. Importantly, gene suppression of HDAC9 concomitantly augmented AGT mRNA and protein levels in cultured PTC, indicating that HDAC9 is a repressor of AGT in PTC. During the development of cardiac hypertrophy, HDAC9 and other class IIa HDACs serve as anti-hypertrophic factors [24, 50]. Taken together, these data suggest that higher levels of HDAC9 in the kidney of females could exert a renoprotective effect via suppressing intrarenal AGT expression as well as its anti-hypertrophic effect.

DNA methyltransferases are also important epigenetic regulators [51]. The results obtained from our PCR demonstrated that renal cortical Smyd1 and Smyd3 mRNA levels in female rats were higher than male rats. Although our PCR could detect Smyd1 in the renal cortex, it has been reported that Smyd1 is a cardiac- and muscle-specific methyltransferase and the expression levels in kidney are very low [52]. Therefore, Smyd1 is unlikely to contribute to establishing the sex difference of intrarenal AGT expression. On the other hand, kidneys express Smyd3 [52]. While the sex difference of renal cortical Smyd3 levels were smaller (1.7-fold in female, compared to male) than the difference of HDAC9 (7.1-fold in female, compared to male), Smyd3 may play a role in lower expression of intrarenal AGT levels in female rats. Further studies will investigate the function of intrarenal Smyd3 in the regulation of intrarenal AGT expression and the development of hypertension and RAS associated kidney injury.

The results of ChIP assays showed that HDAC9 associated with histones is located at the distal region but not the proximal region of the AGT promoter. Although many transcription binding sites have been identified on the proximal region of the AGT promoter [53–55], a recent study showed that the distal region of the AGT promoter also plays an important role in AGT transcription [56]. Thus, deacetylation of a histone on the distal region of the AGT promoter by HDAC9 may suppress binding of transcription factors, which will sustain lower levels of AGT expression in PTC. HDAC9 recruits the monocyte enhancer factor 2 (MEF2) family, converting MEF2 into a transcription repressor [57]. Thus, co-factors might be required for HDAC9-mediated intrarenal AGT suppression. The detailed molecular mechanism will be delineated in further studies.

## Conclusions

In the present study, we demonstrate that levels of intrarenal AGT, but not systemic AGT, were lower in female rats than in males. Female kidneys express higher levels of HDAC9, which suppressed AGT expression by interacting with the AGT promoter in PTC. The demonstration that HDAC9 is an epigenetic suppressing factor involved in the control of AGT expression in renal proximal tubules explains the low levels of intrarenal AGT in females. These findings provide a novel mechanism for regulating intrarenal AGT expression which may help to explain sex disparities in hypertension, associated kidney injury, and the renoprotective effects observed in female subjects.

## Abbreviations

AGT: Angiotensinogen
Ang II: Angiotensin II
HDAC9: Histone deacetylase 9
PTC: Proximal tubular cells
RAS: Renin-angiotensin system
